# Multiple primary melanomas: A literature review

**DOI:** 10.1097/MD.0000000000034378

**Published:** 2023-07-28

**Authors:** Adina Patricia Apostu, Loredana Ungureanu, Salomea Ruth Halmagyi, Ioana Irina Trufin, Simona Corina Șenilă

**Affiliations:** a Clinical Hospital of Infectious Diseases, Cluj Napoca, Romania; b Department of Dermatology, Iuliu Hațieganu University of Medicine and Pharmacy, Cluj Napoca, Romania; c Department of Dermatology, Emergency County Hospital Cluj, Cluj Napoca, Romania.

**Keywords:** epidemiology, incidence, melanoma, multiple primary melanomas, risk factors

## Abstract

Survival rates for melanoma have increased in recent years, a higher number of patients survive after diagnosis, and, therefore, are at an increased risk of developing second primary melanoma. The aim of this literature review is to identify and integrate the incidence rates and other characteristics of multiple primary melanomas. A total of 36 independent studies were included in this review. The incidence of multiple primary melanomas reported ranged from 1.1% to 20.4%. Synchronous melanomas account for 5% to 66% of the reported lesions. The most common site for both first and subsequent melanomas is the trunk. Superficial spreading melanoma is the most common histological type in both first and subsequent primary melanoma. Regarding the mean Breslow index, subsequent melanomas appeared to be thinner than first melanomas. Our review suggests that melanoma patients are at a higher risk of developing a second primary melanoma and long-term surveillance is needed.

## 1. Introduction

Melanoma is the most aggressive form of skin cancer, with a rising incidence worldwide, making it the fifth most commonly diagnosed cancer among both males and females in 2019.^[1]^ In the USA, except for lung cancer, the incidence of melanoma rose faster than any other solid cancer: 2.7% annually from 1986 to 2007.^[2]^ Nowadays, the overall lifetime risk of developing melanoma is one in 50 individuals, compared to 1 in 500 in 1935.^[3,4]^

In Europe, the incidence rate is about 25 new cases of melanoma per 100,000 population, whereas in the US it is 30 new cases per 100,000 populations and 60 cases in Australia. Even if most cases affect the elderly, melanoma stands for the third most common cancer in the adolescents and young adults.^[5,6]^

Most melanomas are diagnosed in an early stage, leading to a 5-year overall survival of 91.8%, but it decreases dramatically to 4% when distant metastases are present. Fortunately, the survival rate improved. In the 1960s’ the mortality rate for melanoma was 60% and now it is 11% albeit the increasing incidence.^[7,8]^

The survival rate for melanoma has increased in recent years due to prevention strategies, health education campaigns and development of new treatments for advanced stages. As a result of prolonged survival, a higher number of patients live longer after diagnosis, and, therefore, are at increased risk of developing a second primary malignancy, especially a second primary melanoma.^[9–12]^

Melanoma is a multifactorial disease, arising from an interaction between genetic susceptibility and environmental factors. However, several risk factors have been described to be associated with developing a second primary melanoma. These include: the presence of dysplastic nevi, family history of melanoma, male gender, early age of onset, fair skin, I/II phototype, severe sunburns, chronic sun exposure, the use of tanning beds and the total number of nevi.^[13–15]^

The survival rate has increased dramatically since Pack et al^[16]^ reported an incidence of multiple primary cutaneous melanoma of 1.28% (16 of 1250) for the first time in 1952. Now, more people are likely to survive the disease and to develop secondary primary cutaneous melanomas.

We performed a narrative review of the current literature on the incidence of multiple primary melanomas in different populations and different periods of time. The aim of this literature review was to identify and integrate the incidence rates and other characteristics of multiple primary melanomas.

## 2. Search strategy

A comprehensive computer-aided search of the databases of published work (PubMed) was done. Furthermore, the reference lists of the included studies were screened. Only studies available in English and in full text were included. The literature search was performed between November 2022 and January 2023. The following keywords were used: multiple primary melanomas, second primary melanomas, second primary neoplasms, melanoma after melanoma in combination with each of the following terms: incidence, epidemiology. Our review included the studies that analyzed both invasive and in situ melanomas, and both synchronous and metachronous lesions. Synchronous lesions were considered as those lesions that developed up to 3 months from the initial diagnosis. Selection bias cannot be ruled out.

## 3. Results

A total of 36 independent studies were included in this review. Most of the patients included in the studies were men, the proportion ranged from 51% to 79%.^[17,18]^ However, some studies reported that females represented the majority (51–67.5%).^[13,19–25]^ The mean age of the population varied between 38.2 and 66 years. The average follow-up period ranged from 1 to 20 years.

Table 11 describes the results found in the studies included in this review. Thirteen countries were represented in the studies, corresponding to 3 continents (Australia, North America, and Europe). The study periods ranged from 5 years^[20,49]^ to 53 years.^[17]^ The largest population included in a study was 65 429.^[17]^

**Table 1:** Summary of the studies included in the review.

Author	Country	Period	MPM/total (%)	Synchronous (%)	Max no of melanomas in one patient	No of primary tumors (mean)
Ariyan et al^[26]^	USA	1983–1993 10	27/423 (6.4%)	8 (25%)	3	NR
Beardmore et al^[27]^	Australia	1963–1972 9	57/1444 (3.9%)	11 (19.2%)	6	NR
Brobeil et al^[18]^	USA	8 yr	101/2600 (4.3%)	67 (66.3%)	NR	NR
Burden et al^[28]^	Scotland	1979–1991 12	45/3818 (1.2%)	12 (26.6%)	6	NR
Burljan et al^[29]^	Croatia	2002–2008	36/991 (3.6%)	11 (30%)	4	NR
Cascinelli et al^[30]^	Italy	1967–1991 24	20/521 (3.8%)	8 (40%)	4	2.1
Chen et al^[17]^	Sweden	1958–2010 52	3599/65 429 (5.5%)	NR	5	NR
De Giorigi et al^[20]^	Italy	2000–2004	40/672 (5.95%)	6 (15%)	NR	NR
Ferrerres et al^[31]^	Spain	1988–2005 17	25/934 (2.6%)	8 (32%)	4	NR
Ferrone et al^[32]^	USA	1996–2002	385/4484 (8.6%)	139 (36%)	NR	2.3
Goggins et al^[33]^	USA	1973–1997	1421/61 245 (2.3%)	313 (22%)	NR	NR
Helgadottir et al^[34]^	Sweden	1960–2004 44	2469/54 884 (4.5%)	NR	NR	NR
Hwa et al^[35]^	USA	2002–2008	61/788 (7.7%)	NR	7	NR
Johnson et al^[36]^	USA	1990–1995	60/1482 (4%)	18 (30%)	7	2.4
Kang et al^[37]^	USA	1965–1989	41/2032 (2%)	16 (39%)	5	NR
Lallas et al^[38]^	Greece	2013–2018	46/977 (4.7%)	NR	6	NR
Manganoni et al^[21]^	Italy	1996–2006	47/1240 (3.7%)	10 (21%)	10	2.4
Menzies et al^[22]^	Ireland	1994–2016	99/2057 (4.8%)	6 (13%)	10	2.5
Moore et al^[39]^	USA	1996–2011	1122/16 570 (6.7%)	NR	>4	NR
Moseley et al^[40]^	USA	1971–1979	38/712 (5.3%)	12 (31%)	8	NR
Muller et al^[41]^	Austria	1968–2015 48	299/1648 (18.1%)	NR	>4	NR
Nashan et al^[23]^	Germany	NR	30/535 (5.6%)	NR	NR	NR
Nosrati et al^[42]^	USA	1985–2013	305/7268 (4.2%)	68 (22%)	13	2.3
Palacios Diaz et al^[43]^	Spain	2014–2022	58/646 (8.9%)	20 (34%)	5	2.2
Pastor-Tomas et al^[14]^	Spain	2000–2015	55/1447 (3.8%)	NR	NR	NR
Savoia et al^[19]^	Italy	1974–1997	49/2470 (2%)	21 (43%)	6	NR
Scheibner et al^[44]^	Australia	1951–1980	90/3128 (2.9%)	Aprox. 33%	5	NR
Schmid et al^[45]^	Germany	1977–1992	152/4597 (3.3%)	58 (37%)	NR	NR
Siskind et al^[46]^	Australia	1982–1990	221/1083 (20.4%)	13 (5%)	9	NR
Stam-Posthuma et al^[13]^	Netherlands	1983–1995	56/820 (6.8%)	13 (23%)	9	2.8
Titus-Ernstoff et al^[47]^	USA	NR	27/354 (8.0%)	8 (29%)	3	2.1
Ungureanu et al^[24]^	Romania	2004–2020	26/699 (3.7%)	13 (50%)	4	2.3
van der Leest et al^[48]^	Netherlands	1989–2008	1840/57 465 (3.2%)	NR	NR	NR
Villani et al^[49]^	Italy	2014–2018	64/773 (8.2%)	NR	6	NR
Wassberg et al^[25]^	Sweden	1958–1988	243/20,354 (1.1%)	NR	NR	NR
Wiener et al^[50]^	USA	1998–2012	5960/152,811 (3.9%)	1743 (29%)	NR	NR

MPM = multiple primary melanomas, NR = not reported.

The incidence of multiple primary melanomas (MPM) reported ranged depending on the study from 1.1%^[25]^ to 20.4%.^[46]^ The highest incidence of 20.4% was reported in Australia, while the highest incidence in Europe and North America was 18.1% in Austria^[41]^ and 8.6% in the US respectively.^[32]^

Synchronous melanomas represented 5%^[46]^ to 66%^[18]^ of the reported lesions. The highest number of primary tumors in one individual was 13,^[42]^ and the mean number of primary tumors per individual ranged from 2.1 to 2.8.^[13]^

Table 2 describes the tumor characteristics of the first melanomas, compared with the characteristics of subsequent melanomas. The most common site for both first and second primary melanomas was the trunk. Superficial spreading melanoma was the most common histological type in both first and second primary melanoma.

**Table 2:** Comparison of tumor characteristics of the first and second primary invasive melanoma.

Author	Anatomic site		Breslow index (mm) (mean)		Histologic type	
	First melanoma	Second melanoma	First melanoma	Second melanoma	First melanoma	Second melanoma
Ariyan et al^[26]^	Trunk	Trunk	NR	NR	NR	NR
Beardmore et al^[27]^	Trunk	Trunk	NR	NR	NR	NR
Brobeil et al^[18]^	NR	NR	2.27	0.90	NR	NR
Burden et al^[28]^	NR	NR	2.1	1.2	NR	NR
Burljan et al^[29]^	Trunk	Trunk	3.1	1.3	SSM	SSM
Cascinelli et al^[30]^	NR	NR	NR	NR	NR	NR
Chen et al^[17]^	Limbs	NR	NR	NR	SSM	NR
De Giorigi et al^[20]^	NR	NR	0.51	0.64	NR	NR
Ferrerres et al^[31]^	Trunk	NR	1.41	0.34	SSM	SSM
Ferrone et al^[32]^	Trunk	Extremities	1.2	0.4	NR	NR
Goggins et al^[33]^	NR	NR	NR	NR	NR	NR
Helgadottir et al^[34]^	Trunk	NR	NR	NR	NR	NR
Hwa et al^[35]^	Trunk	Trunk	NR	NR	SSM	SSM
Johnson et al^[36]^	Trunk	NR	0.98	0.90	NR	NR
Kang et al^[37]^	Trunk	Trunk	1.21	0.51	SSM	SSM
Lallas et al^[38]^	NR	NR	1.92	0.49	NR	NR
Manganoni et al^[21]^	Trunk	Trunk	1.31	0.66	NR	NR
Menzies et al^[22]^	Lower limbs	Lower limbs	1.21	0.36	SSM	SSM
Moore et al^[39]^	Head and neck	Head and neck	1.05	0.83	SSM	SSM
Moseley et al^[40]^	Trunk	Extremities	NR	NR	NR	NR
Muller et al^[41]^	NR	NR	NR	NR	NR	NR
Nashan et al^[23]^	NR	Lower limbs	NR	0.59	NR	NR
Nosrati et al^[42]^	NR	NR	2.2	1.1	NR	NR
Palacios Diaz et al^[43]^	Trunk	Trunk	1.54	0.65	SSM	SSM
Pastor-Tomas et al^[14]^	Trunk	Trunk	NR	NR	SSM	SSM
Savoia et al^[19]^	Trunk	Trunk	2.2	0.64	SSM	SSM
Scheibner et al^[44]^	Trunk	Trunk	NR	NR	SSM	SSM
Schmid et al^[45]^	Trunk	Trunk	1.6	0.7	SSM	NR
Siskind et al^[46]^	Trunk	Trunk	0.82	0.85	SSM	SSM
Stam-Posthuma et al^[13]^	Trunk	Trunk	1.11	0.9	SSM	SSM
Titus-Ernstoff et al^[47]^	NR	NR	NR	NR	NR	NR
Ungureanu et al^[24]^	NR	NR	NR	NR	SSM	SSM
van der Leest et al^[48]^	Trunk	Trunk	NR	NR	SSM	SSM
Villani et al^[49]^	NR	NR	0.97	0.39	NR	NR
Wassberg et al^[25]^	NR	NR	NR	NR	NR	NR
Wiener et al^[50]^	Trunk	NR	NR	NR	NR	NR

NR = not reported, SSM = superficial spreading melanoma.

Regarding the mean Breslow index, subsequent melanomas appeared to be thinner than first melanomas, except for 2 studies, in which the Breslow index was higher in subsequent primary lesions.^[20,46]^ The largest decrease in the mean Breslow index was reported by Buljan et al^[29]^ – 1.8 mm.

## 4. Discussion

The studies showed that melanoma patients have a higher risk of developing subsequent primary melanoma, emphasizing the increasing incidence of multiple primary melanomas in the general population in recent years. The incidence of multiple primary melanomas ranged from 1.1% to 20.4% depending on population, study design and follow-up length.

Comparison between continents showed, as expected due to the decreasing UV levels, the highest incidence of multiple primary melanomas in Australia – 20.4%^[46]^ and the lowest in Europe – Sweden 1.1%.^[25]^This is similar to the incidence rates of melanoma for these regions at different geographic latitudes. Unfortunately, only a limited number of studies covering just 3 continents were available to be included in this review, no data being available from Asia, Africa or South America.

The longest average follow-up period was reported by Muller et al,^[41]^ Savoia et al,^[19]^ Siskind et al,^[46]^ and Goggins et al,^[33]^ accounting for 10.1 years, 12 years, 16.5 years, and 20 years respectively. Interestingly, Muller et al^[41]^ and Siskind et al^[46]^ reported the highest incidence rates of multiple primary melanomas of 18.14% and 20.4%, which can be explained by the long follow-up period. On the other hand, Savoia et al^[19]^ and Goggins et al^[33]^ reported an incidence rate of MPM of 2% and 2.32% respectively. One explanation for this finding might be the older study periods when compared to Siskind et al and Muller et al and the country where the studies were conducted. (Spain and USA compared to Austria and Australia).

Most studies reported a male preponderance, although some studies commented on the opposite sex ratio in their series^.[13,19–25]^ Female patients are associated with a higher awareness,^[48]^ are more compliant with follow-up recommendations and have lower tumor thickness for their second melanoma when compared to men.^[20]^ Females might be affected more than men because female patients have better prognosis and longer survival and therefore have more time to develop a second melanoma.

Male gender is considered a risk factor for developing a second melanoma.^[42]^ Besides gender, personal history of melanoma, positive family history of melanomas and presence of dyspastic nevi are other risk factors for multiple primary melanomas.^[29,35,37]^

Titus-Ernstoff et al^[47]^ showed that the presence of 3 or more dysplastic nevi was associated with more than a 4-fold risk of MPM. Similarly, Ferrone et al found that patients with positive family history of melanoma or with dysplastic nevi have a much higher risk of developing second primary melanoma and it is usually diagnosed 10 years earlier than in the general population.^[32,51]^

Moreover, several studies suggested that there could be a genetic predisposition for MPM and that gene CDKN2A and CDK4 could be involved.^[52–54]^ CDKN2A gene mutations are rare in the general populations, but they are found in 5% to 20% of familial and multiple primary melanoma patients. Around 80% of CDKN2A mutation carriers will develop multiple primary melanomas.^[55–58]^

Regarding the age, early age of onset is considered a risk factor for multiple primary melanomas, but many studies reported the opposite – MPM patients were older than the single primary melanoma (SPM) cohort and patients aged > 60 years at the time of diagnosis of the first primary melanoma have a greater risk of developing second primary melanoma.^[33,35,39,42,43,59]^ Furthermore, Menzies et al^[22]^ found no significant difference in age between the 2 groups (64 years vs 66 years, respectively).

With more than 100 common melanocytic nevi and more than 50 cherry angiomas, red and blonde hair was associated with an increased risk of developing subsequent melanoma in a study conducted by Pastor-Tomas et al^[14]^ While light hair color and the presence of multiple melanocytic nevi are well known risk factors for developing melanoma^[46,60,61]^ the association between multiple cherry angiomas and the increased risk of developing subsequent melanoma was an unexpected finding.

Hwa et al^[35]^ examined tumor mitotic rate and compared SPM with MPM. They found that there was no significant difference in tumor mitotic rate between patients with single primary melanomas and multiple primary melanomas. On the other hand, 2 independent studies^[43,62]^ found that second primary melanomas are more prone to develop histological regression than first melanomas. The regression phenomenon can be explained by an immunologic surveillance response, following an “immunization effect” from being exposed to previous melanomas.^[63]^ Also, the higher rate of histological regression may explain the lower Breslow index in subsequent melanomas.

Most patients developed 2 melanomas, the mean number of primary tumors per patient ranged from 2.1^[47]^ to 2.8.^[13]^ The maximum number of melanomas found in 1 patient ranged from 3^[26,47]^ to 13.^[42]^ A case in the literature reported as many as 48 melanomas in 1 patient.^[64]^ Up to 66% of reported lesions appeared synchronously. This finding supports the importance of comprehensive skin examination in melanoma patients, not only at the first visit, but also during all follow-ups. Brobeil et al^[18]^ highlighted the consequence of receiving regular skin examinations in melanoma patients. In their study, 93% of secondary primary melanomas were observed by a physician. On the other hand, patients with self-detected melanomas had a higher Breslow index than patients with melanomas that were detected by a dermatologist.^[65]^ Superficial spreading melanoma was the most frequent histologic subtype reported for both first and second primary melanomas, followed by nodular melanoma and lentigo maligna subtypes. Ungureanu et al^[24]^ found that nodular melanoma was predominantly seen as first melanoma, while Moore et al^[39]^ reported that nodular melanoma represented less than 2% of all subsequent tumors. As for lentigo maligna, Palacio-Diaz et al^[43]^ reported an increasing rate of this histologic subtype in subsequent melanomas probably due to chronic UV radiation exposure. Another study found that in only half of the patients the histologic type of the first melanoma coincided with the second, superficial spreading melanoma being the subtype with the highest concordance.^[14]^

The most common lesion site was the trunk, although some studies reported the legs as being the most common site in women.^[13,31,36,37]^ According to the studies, second primary melanomas do not necessarily develop in the same anatomical region as the initial lesions. Several studies reported that in about 50% of the patients second primary melanomas were located in a different part of the body.^[24,31,35,43,47]^ Stam-Posthuman et al,^[13]^ Pastor-Tomas et al,^[14]^ and Kang et al^[37]^ found that around 70% of second primary melanomas developed in a different anatomical region than the first melanoma. Nonetheless, Helgadottir et al^[34]^ found that women with a first melanoma in the head and neck area and men with a first melanoma of the trunk are prone to develop a second melanoma in the same area. A possible explanation for this finding can be that patients with melanoma in the more chronically UV-exposed areas are more likely to develop a second melanoma on the same area of the body, when compared with those that have first melanoma in the more intermittently exposed body sites. These findings show that there is no overall pattern in the distribution of MPM and complete skin examination should be performed at every visit. Subsequent melanomas had a lower mean Breslow index than first melanomas. One possible explanation for this phenomenon is the early detection due to rigorous controls and adherence to regular follow-up. An alternative explanation is a less aggressive tumor biology in patients with MPM versus SPM.^[32]^ Some studies showed a better prognosis in patients with MPM compared to patients with SPM. A possible hypothesis for the increased survival of patients with MPM is the immune response in patients with MPM to each subsequent melanoma, a so-called “immunization effect.”^[40,44,63,66]^ Moreover, an overall decrease in tumor thickness has been reported in several countries in the past few decades.^[67]^ Nevertheless, 2 studies showed a higher thickness for subsequent melanomas.^[20,46]^ The explanation for this finding can be the lack of patient adherence to regular follow-ups. Di Giorgi et al^[20]^ reported that 67.5% of patients attended periodical clinical follow-up examinations and performed regular self-examinations and found that women were more compliant with follow-up recommendations compared to men (74% vs 54%). Moreover, they compared the mean Breslow thickness for those who closely adhered to follow-up recommendations with those who did not attend follow-ups and found significantly higher values in the latter (1.22 mm vs 0.36 mm). The biological differences between first and subsequent melanomas also cannot be ruled out.

Most studies reported a higher risk of subsequent primary melanoma in the first 2 years after the first primary melanoma and remains higher in the first 5 years. However, several studies reported the occurrence of second primary melanoma 2 or 3 decades after the first melanoma.^[19–23,27,29,40,42,43]^ Scheibner et al^[44]^ reported the occurrence of subsequent primary melanoma 41 years after the first diagnosis, which might indicate that the risk remains stable and does not diminish with time.

## 5. Conclusion

Due to cost and time concerns, screening cannot include the entire population and high risk individuals need to be identified. Melanoma patients should be advised about the high risk of developing subsequent melanoma and motivated to perform self-examinations, avoid sunburns and tanning and see a specialized physician in case of a suspicious lesion. They also need to have full skin examination annually, performed by a trained physician in order to detect not only metastases, but also second melanomas early. Frequency and duration of follow-up recommendations in melanoma patients remain controversial, but considering that a second melanoma could develop even several decades after the initial diagnosis, lifelong follow-ups should be recommended.

## Author contributions

**Conceptualization:** Adina Patricia Apostu, Loredana Ungureanu.

**Data curation:** Adina Patricia Apostu, Salomea Ruth Halmagyi, Ioana Irina Trufin.

**Methodology:** Adina Patricia Apostu, Loredana Ungureanu.

**Software:** Adina Patricia Apostu, Salomea Ruth Halmagyi, Ioana Irina Trufin.

**Supervision:** Loredana Ungureanu.

**Validation:** Adina Patricia Apostu, Loredana Ungureanu.

**Visualization:** Adina Patricia Apostu.

**Writing – original draft:** Adina Patricia Apostu, Salomea Ruth Halmagyi, Ioana Irina Trufin.

**Writing – review & editing:** Adina Patricia Apostu, Loredana Ungureanu.
